# In-Fiber Closed Cavity Interferometric High-Resolution Aqueous Solution and Alcohol Gas Refractometer

**DOI:** 10.3390/s19102319

**Published:** 2019-05-20

**Authors:** Lijun Li, Tianzong Xu, Yinming Liu, Zhaochuan Zhang, Qian Ma, Zhihui Shi, Weikang Jia, Jianhong Sun, Fei Yu, Paulino Mba Ndong Mangue

**Affiliations:** 1 College of Electronic and Information Engineering, Shandong University of Science and Technology, Qingdao 266590, China; zzc0524@126.com (Z.Z.); qianma@sdust.edu.cn (Q.M.); 17860763071@163.com (Z.S.); wkjia6868@163.com (W.J.); sunjianhong815@163.com (J.S.); m15764246412_2@163.com (F.Y.); pndongmangue@gmail.com (P.M.N.M.); 2 College of Electrical Engineering and Automation, Shandong University of Science and Technology, Qingdao 266590, China; xutianzong@sdust.edu.cn; 3 State Key Laboratory of Mining Disaster Prevention and Control Co-founded by Shandong Province and the Ministry of Science and Technology, Shandong University of Science and Technology, Qingdao 266590, China; 4 Library, Shandong University of Science and Technology, Qingdao 266590, China; liuyinming@sdust.edu.cn

**Keywords:** optical fiber sensor, refractive index sensor, graphene filled cavity, alcohol gas, low concentration aqueous solution

## Abstract

An optical fiber interferometric refractometer for alcohol gas concentration and low refractive index (RI) solution (with 1.33–1.38 RI range) measurement is theoretically and experimentally demonstrated. The refractometer is based on a single-mode thin-core single-mode (STS) interferometric structure. By embedding a suitably sized air cavity at the splicing point, high-order cladding modes are successfully excited, which makes the sensor more suitable for low RI solution measurement. The effect of the air cavity’s diameter on the sensitivity of alcohol gas concentration was analyzed experimentally, which proved that RI sensitivity will increase with an enlarged diameter of the air cavity. On this basis, the air cavity is filled with graphene in order to improve the sensitivity of the sensor; and the measured sensitivity of the alcohol gas concentration is −1206.1 pm/%. Finally, the characteristics of the single-cavity structure, graphene-filled structure and double-cavity structure sensors are demonstrated, and the linear RI sensitivities are −54.593 nm/RIU (refractive index unit), −85.561 nm/RIU and 359.77 nm/RIU, respectively. Moreover, these sensor structures have the advantages of being compact and easily prepared.

## 1. Introduction

Optical fiber interferometric refractive index (RI) sensors such as in-fiber Mach-Zehnder, Michelson, Fabry-Pérot and Sagnac refractometers are attracting increasing interest in the context of chemical and biochemical sensing applications due to their high sensitivity, well established immunity to electromagnetic interference, small size and their ability to be remotely operated [1,2,3,4]. In these existing refractometers, cores mismatch sensing structures are usually used, and the resolution of this kind of sensor is generally 10^−3^~10^−4^ in the range of 1.3 to 1.4 RI and 10^−4^~10^−5^ in the range of 1.4 to 1.5 RI according to either low and high concentrations of aqueous solution, respectively [1,2,5]. Due to the inherent effective refractive index of the optical fiber, higher-order cladding modes with a lower effective refractive index in these sensing structures, which are required for sensing a low concentration of aqueous solution, and even gas, is difficult to excite. In some RI sensing applications, especially biosensors, there is an increasing need to detect the resolution of the low concentration solution, such as in the medical community and for biomedical sensing measurement [6,7,8,9,10]. Therefore, it is desirable to improve the RI sensing sensitivity of a low concentration solution, and even the gas, with a lower refractive index. In order to increase the sensitivity, some post-processing of the sensing part of the optical fiber, such as tapering and etching [11,12,13]. These methods were used to improve the resolution through reducing the optical fiber cladding diameter in order to excite more higher order cladding modes with a lower effective refractive index. Further resolution improvement was achieved by exposing the optical fiber core through fabricating open-cavity in the fiber [4,14,15]. However, in these existing refractometers, an open-cavity and weak mechanical properties are two main problems that need to be solved. The sensitizing methods of abrupt taper, etching cladding, femtosecond laser cavity fabrication and hollow fiber cavity mean that the sensors can be easily damaged and disturbed due to their thin and micro-scale. Furthermore, the open-cavity sensing structure can also present a challenge with respect to cleanness, thus affecting the accuracy and repeatability of the sensor.

In this paper, a kind of high-resolution closed cavity fiber interferometric refractometer for alcohol gas and low concentration liquid is proposed. Refractive index sensing characteristics of single-cavity, graphene-filled cavity and double-cavity refractometers are experimentally investigated and theoretically simulated. The simulation results show that the high-order cladding modes with a lower effective refractive index can be excited by introducing a suitable size of closed cavity. For alcohol gas concentration sensing, a single-cavity interferometric in-fiber refractometer with different cavity sizes is experimentally demonstrated, of which the maximum sensitivity can reach −983.1 pm/%. Then, a graphene filled cavity RI sensor with the same cavity size is also studied, its sensitivity is significantly improved to −1206.1 pm/% due to the fact that graphene allows more light energy into the cladding. For a low concentration solution with 1.33 to 1.38 RI range, refractometers with single-cavity, graphene-filled cavity and double-cavities are experimentally investigated, and the experimental results show that their sensitivities are −54593 pm/RIU (refractive index unit), −85561 pm/RIU and 359,770 pm/RIU, respectively. The maximum sensitivity corresponds to $5.6\times 10^{-5}$ RI resolution.

## 2. Principles and Fabrication

### 2.1. Operating Principles and Simulation

**Figure 1:**
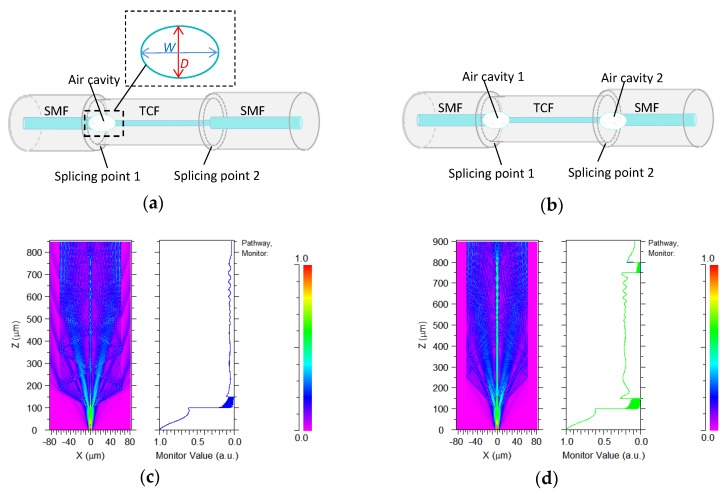
Schematic diagram of (**a**) single-cavity interferometric sensor structure and (**b**) double-cavities interferometric sensor structure, respectively. Simulation of light propagation within (**c**) single-cavity sensor and (**d**) double-cavities sensor.

In our experiments, the schematic diagrams of the RI sensors are shown in Figure 1, in which a section of thin-core fiber (TCF) is spliced between two single mode optical fibers (SMF) to compose a SMF-TCF-SMF (STS) structure, and one or two air cavities are embedded in the optical fiber cladding at splicing points between the SMF and TCF, as shown in Figure 1a,b, respectively. Figure 1c illustrates the simulation of the light propagation within a single-cavity sensor. The length of the cavity is indicated by *W*, and the longitudinal diameter of the cavity is denoted by *D*. From Figure 1c, it can be found that the light injects into the input-SMF and then interferes in the air cavity. After the cavity, lots of cladding modes are stimulated due to the core to core mismatch at the first splicing point, which is produced by the air cavity. As evanescent fields, cladding modes can carry the refractive index information of the surrounding materials. At the second splicing point between the TCF and SMF, these cladding modes can be partly returned to the fiber core and induce interference with the core modes. In Figure 1c, the interference light in the core of the output SMF can be observed obviously. Therefore, the surrounding RI can be obtained by measuring the interference spectrum of the sensor. Based on this operating principle, this sensor can be seen as an in-fiber Mach-Zehnder (M-Z) interferometer and its output spectrum expression can be written as [1]

(1) $$I_{out}=I_{core}+{\sum_{m}I}_{clad}^{m}+\sum_{m}2\sqrt{I_{core}{I^{m}}_{clad}}\cos\left[\frac{2\pi \left(n_{eff,core}-n^{m}{}_{eff,clad}\right)L}{\lambda }\right]$$

where, $I_{core}$ and $I^{m}{}_{clad}$ indicates the light intensity of the core and m-order cladding modes in the core of the output SMF, respectively. Moreover, $n_{eff,core}$ and $n^{m}{}_{eff,cladd}$ represents the effective RIs of the core and m-order cladding modes, respectively. In addition, L denotes the geometry length of the TCF, while λ represents the wavelength of the propagating light in vacuum.

From Equation (1), when the interference transmission spectrum reaches its minimum, the intensity dip wavelength of the *m* order can be written as follows:

(2) $$\lambda_{m}=\frac{2\left(n_{eff,core}-n^{m}{}_{eff,clad}\right)L}{2m+1}$$

In the double-cavity sensor, light propagation in the sensing structure is shown in Figure 1d. We can find that the light injects into the input-SMF and induces interference in the first cavity at the first splicing point. Similar to the single-cavity structure, lots of cladding modes are stimulated by the first cavity, and the input light is divided into two parts, which pass through the core and cladding of the TCF, respectively. Cladding modes carry the RI information of the surrounding and are recoupled into the second air cavity at the second splicing point. As light meets the cavity, some of the light energy ($I_{1}$) directly passes through the cavity due to the low reflectivity of the inner wall of the cavity, and the other part of the light energy ($I_{2}$) emits out of the cavity after repeated reflection, which forms the interference spectrum. Therefore, this sensing structure can be seen as a low fineness Fabry-Pérot (F-P) cavity, in which light travels in the core as paraxial light, so it can be considered as normal incidence. The expression of the output interference light intensity of this sensor can be written as:

(3) $$\begin{array}{l} I_{out}=I_{1}+I_{2}+\sum_{m}2\sqrt{I^{m}{{}_{1}}_{,clad}I^{m}{}_{2,clad}}\cos\left(\frac{4\pi }{\lambda }n^{m}{}_{eff,clad}W\right)+2\sqrt{I_{1}{}_{,core}I_{2,core}}\cos\left(\frac{4\pi }{\lambda }n_{eff,core}W\right)+\\ \sum_{m}2\sqrt{I_{\mathrm{core}}I^{m}{}_{clad}}\cos\left[\frac{2\pi }{\lambda }\left(n_{eff,core}-n^{m}{}_{eff,clad}\right)W\right] \end{array}$$

where, $I_{1,core}$ and $I_{2,core}$ are direct and reflected pass core modes of light intensity, respectively; $I^{m}{}_{1,clad}$ and $I^{m}{}_{2,clad}$ are direct and reflected pass cladding modes of light intensity, respectively.

From this equation, consider the cladding modes in this F-P cavity, the interference dip wavelength according to the m-order cladding mode is:

(4) $$\lambda_{m}=\frac{4n^{m}{}_{eff,clad}W}{2m+1}$$

**Figure 2:**
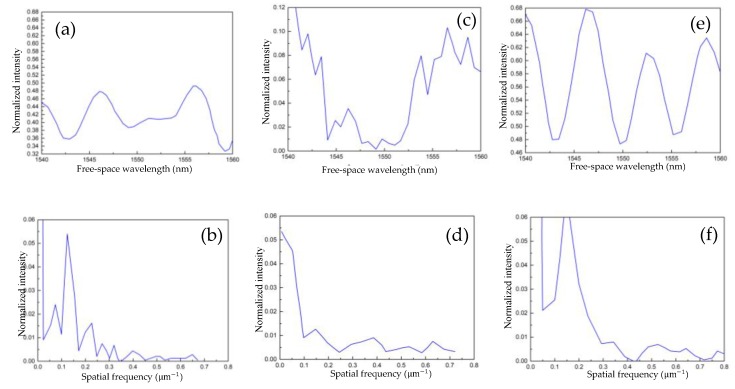
(**a**) Simulation results of transmission interference spectrum and its (**b**) Fourier transformed spectrum of STS M-Z interferometer. (**c**) One air cavity (W = 100 μm and D = 8.4 μm) M-Z interferometer simulation transmission and its (**d**) Fourier transformed spectrum. (**e**) One air cavity (W = 20 μm and D = 80 μm) M-Z interferometer simulation transmission and its (**f**) Fourier transformed spectrum.

This output spectrum is composed of core and cladding modes themselves, and their mutual interference. In order to stimulate higher-order cladding modes with a lower effective RI, an air cavity is embedded in the optical cladding of the in-fiber sensor. The difference of the optical field in the thin core fiber of Figure 1c,d is mainly due to the large optical power loss of the two cavity sensor, which can be found from the comparison of the monitor power values of these two figures. In our simulation, the RI of the core and cladding of the SMF with 1.4544 and 1.45 and TCF with 1.4641 and 1.45 are adopted, respectively. The diameter ratio of core/cladding of SMF with 8 μm/125 μm and TCF with 5 μm/125 μm, respectively. By using the finite difference beam propagation method, the interference spectra of the different sensors of cavity-free (shown as Figure 2a) and one air-cavity with cavity size of W = 100 μm, D = 8.4 μm (shown as Figure 2c) and W = 20 μm, D = 80 μm (shown as Figure 2e) are simulated. Their spatial frequency spectra, which is expanded according to (1λ), can be obtained through a Fourier transform of their interference spectra, which are shown in Figure 2b,d,f. The effective refractive index of the dominant cladding modes can be obtained through analyzing its spatial frequency spectrum. The high spatial frequency of the high order cladding mode corresponds to the low effective refractive index. By comparing Figure 2b,d, it can be found that the higher order cladding mode can be introduced through the inner air cavity of the core. The simulation results show that the cladding mode expands to a higher order with the length of the cavity increasing, but the increase of cavity length also leads to a greater loss and deterioration of the interference spectrum, which is shown as Figure 2c,d. Through simulation, we also find that some higher cladding modes can be further introduced by enlarging the air cavity diameter, which not only reduces the loss of optical power, but also optimizes the spectrum of the sensor, which is shown as Figure 2e,f.

### 2.2. Sensing Head Fabrication

**Figure 3:**
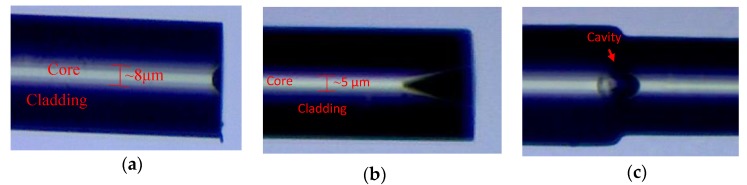
Microscopic photos of half cavity at the fiber tip of (**a**) SMF, (**b**) TCF and (**c**) closed air cavity in the fiber core.

The microcavity in the fiber core is fabricated by the hydrofluoric acid etching method [16,17]. Due to the corrosion rate of Germanium doped fiber core being higher than that of fused quartz cladding, a micron-sized cavity can be obtained inner the core as corroding the cleaved fiber end for a period of time, and the size of the microcavity can be designed through controlling the corrosion time. The microscopic photos of the cavity are illustrated in Figure 3. In our experiments, two of the same open microcavities are fabricated in the cores of an SMF and a TCF fiber tip, respectively, which is shown in Figure 3a,b. Then, these two fibers are spliced face to face by a fusion splicer with a weak discharge intensity mode. The microscopic photo of the final embedded fiber microcavity is shown in Figure 3c. Due to the rapid hot melting and cooling of the silica fiber core, with the thermal expansion of air in the cavity, the result is the formation of a smooth surface of an ellipsoidal microcavity. In these sensors, the thin core fiber (TCF) is inserted in the middle of the sensing structure, which can effectively improve the stability of the sensor due to its bending insensitive performance. In the one-cavity M-Z sensor, the length of the TCF is closely related to the spectrum and sensitivity of the sensor [1,18,19]; therefore, this needs to be taken into consideration in the fabrication of the sensor. In our experiment, the TCF length is 30 mm. In order to further improve the sensitivity of the sensor, graphene film is covered in the interior cavity. The graphene is single-layered with greater than 99% purity, 0.6-to-1 nm thickness, 0.5-to-5 μm slice diameter, and 1000 to 1217 m^2^/g specific surface area. The graphene is ultrasonically dispersed in pure dimethylformamide (DMF) solution for several hours and then centrifuged for 5 min in order to remove air bubbles from the solution. Then, the optical fiber tips with a microcavity are immersed in the graphene solution for several minutes. Then, the fiber tips are removed to a temperature-controlled box within 40 °C. Finally, a layer of graphene film is formed on the surface of the microcavity. 

## 3. Experimental Results

### 3.1. Alcohol Gas Concentration Sensing

**Figure 4:**
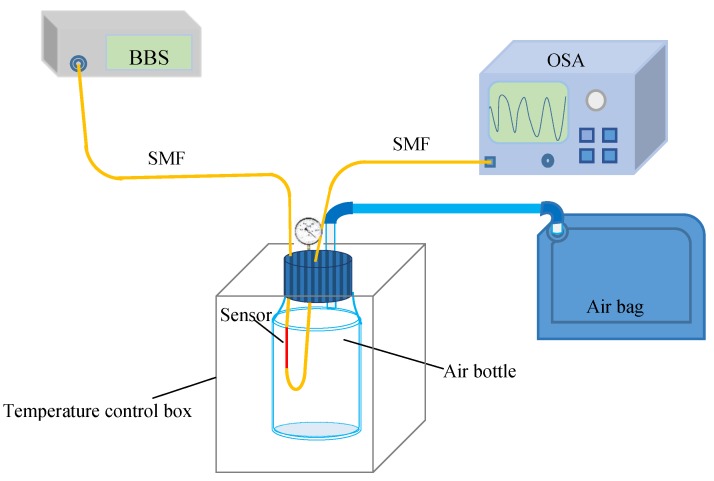
The schematic diagram of the alcohol vapor concentration sensing setup.

The schematic diagram of the alcohol gas concentration sensing system is set up in Figure 4. Light from a broad band source (BBS) injects into the sensor from its one-end spliced SMF, and the transmission spectrum of the sensor is sent to an optical spectrum analyzer (OSA) from a spliced SMF at another end. The sensor is placed in an alcoholic gas bottle, which is placed in a 40 °C temperature control box. In order to ensure that the air pressure in the bottle is the same with as the surrounding atmosphere, the bottle is tubed to an exhausted large-volume empty soft bag.

**Figure 5:**
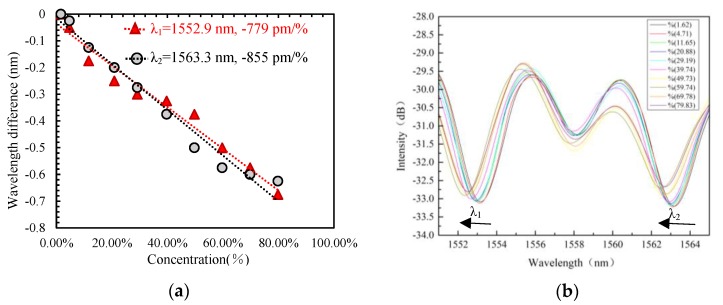
(**a**) Linear fittings of two different wavelengths shift with alcohol gas concentration. (**b**) Superposition spectrum of the sensor at different alcohol gas concentrations.

In our experiment, the concentration of the alcohol gas is increased from 1% to 80%, with which the dip wavelength of the senor (with W = 23.5 and D = 16.2 cavity size) interference spectrum presents a blue-shift. The linear fitting curves of the experimental results and superposition spectrum of the sensor corresponding to different alcohol gas concentrations are shown in Figure 5a,b, respectively. In order to compare the sensitivity between different dip wavelengths of the sensor, a wavelength difference between the sensing wavelength and its initial wavelength is adopted, which is shown in Figure 5a. From their linear fitting curves, it can be found that according two different dip wavelengths, their sensitivities of wavelength difference versus gas concentration are −779 pm/% and −855 pm/%, respectively; and the longer wavelength assumes a higher sensitivity, primarily because the longer wavelength corresponds to the higher order cladding mode with a larger mode field area. 

**Figure 6:**
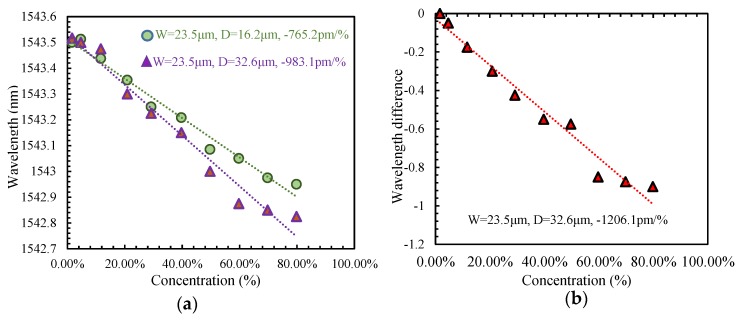
Linear fittings between dip wavelength and alcohol concentration of (**a**) two sensors with different cavity diameter, respectively. (**b**) Linear fitting between dip wavelength difference and alcohol concentration of graphene-filled sensor.

In order to understand the relationship between the sensitivity of the sensor and its cavity diameter, two single-cavity sensors with different diameter with 16.2 μm and 32.6 μm, and the same cavity length as 23.5 μm are experimentally investigated, respectively. With the alcohol gas concentration increasing, dips at the 1543.5 nm initial wavelength of the interference spectrum of two sensors all present blue shift with different sensitivity, their linear fitting curves are shown in Figure 6a.

From Figure 6, it can be found that the sensitivity of this kind of closed cavity refractometer can be improved from −765.2 pm/% to −983.1 pm/% through enlarging the cavity diameter from 16.2 μm to 32.2 μm, which is the same as the results of our theoretical simulations. After this, a graphene film is coated on the surface of the inner cavity to compose a graphene-filled closed cavity Mach-Zehnder interferometric sensor. Its alcohol gas concentration sensing characteristic is also experimentally investigated, of which the linear fitting curve is shown in Figure 6b. The dip wavelength blue shift is also observed and the sensitivity of the sensor is significantly improved to −1206.1 pm/%, which is mainly due to the graphene having a higher refractive index than air and it covering the inner surface of the cavity, which will cause the energy of the evanescent field to concentrate on the surface of the cavity, thus increasing the contact area between the light field and the surrounding [20,21,22].

### 3.2. Low Concentration Solution Sensing

**Figure 7:**
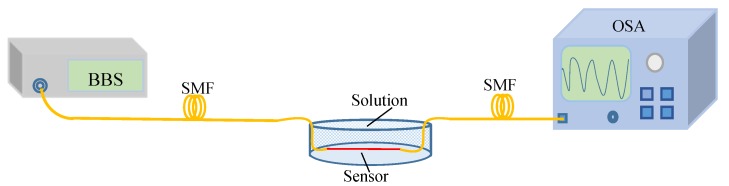
Solution RI sensing experimental setup.

A low concentration solution RI within 1.33 to 1.38 range sensing characteristics of the single-cavity, graphene-filled cavity and double-cavity sensors are also experimentally demonstrated. The experimental setup is shown in Figure 7. The broad band light emits from the BBS and injects into the sensor through the SMF; the sensor is immersed in the solution, and an OSA with 0.02 nm resolution is used to observe the transmission spectrum of the sensor. 

**Figure 8:**
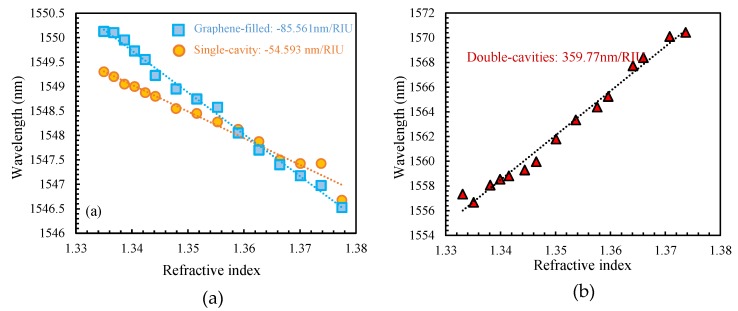
Linear fitting curves between dip wavelength and solution RI of (**a**) single-cavity and graphene-filled single-cavity sensor, and (**b**) double-cavities sensor, respectively.

Linear fitting curves of single-cavity and graphene filled single-cavity sensor are shown in Figure 8a. From this figure, it can be found that dip wavelengths assume a blue shift with the RI increasing from 1.33 to 1.38, and the sensors have −54593 pm/RIU sensitivity in the single-cavity sensor and −85561 pm/RIU sensitivity in the graphene filled single-cavity sensor, respectively. The linear fitting curve of the double-cavity sensor is shown in Figure 8b, from which it can be found that the dip wavelength is red-shifted with the RI increase with 359,770 pm/RIU sensitivity corresponding to a $5.6\times 10^{-5}$ RI resolution.

## 4. Conclusions

In conclusion, the characteristics of a kind of high-resolution refractometer, based on an in-fiber closed cavity interferometric structure, and its response to the alcohol gas concentration and a low concentration solution are theoretically analyzed and experimentally verified. By embedding the air cavity at the splicing point, high-order cladding modes are successfully excited which are more sensitive to RI close to 1.33 of lower concentration solution and even lower refractive index with respect to gas. The effects of the air cavity diameter, graphene filling, and number of air cavities on sensor performance are simulated and experimentally demonstrated. From this research, it is found that increasing the cavity length will significantly increase the loss of power and is also unfavorable to the sensitivity of the sensor. Furthermore, increasing the diameter of the cavity can effectively improve the sensitivity of the sensor and reduce power loss. Meanwhile, the formation of a graphene film in the cavity surface can also effectively improve the sensitivity of the sensor. By selecting the appropriate air cavity diameter, the experimental results show that the filling of graphene can increase the alcohol gas concentration sensitivity from −983.1 pm/% to −1206.1 pm/% and increase the RI sensitivity from −54,593 pm/RIU to −85,561 pm/RIU. Then, a double-cavity structure was analyzed and verified, and the RI sensitivity was significantly increased to 359,770 pm/RIU. Because of its compact structure, good mechanical properties, easy fabrication and high sensitivity to low RI solutions, this sensor shows good potential for aqueous chemical and biological solution detection.
